# Work-Related Factors and Their Influence on Body Mass Index: A Retrospective Cohort Study in the French Tertiary Sector

**DOI:** 10.3390/jcm14207399

**Published:** 2025-10-20

**Authors:** Antoine Soprani, Adrien Soprani, Viola Zulian, Antonio Iannelli, Sergio Carandina

**Affiliations:** 1 Centre Médico-Chirurgical de l’Obésité (CMO), Hôpital Privé Geoffroy Saint Hilaire, Ramsay Sante, 75005 Paris, France; antoinesoprani@gmail.com; 2 Médecin de Travail, Efficience Santé au Travail, 5 rue Antoine Bourdelle, 75015 Paris, France; adrien.soprani@gmail.com; 3 ELSAN, Clinique Saint Michel, Centre de Chirurgie de l’Obésité (CCO), 83100 Toulon, France; zulian.viola@gmail.com (V.Z.); iannelli.a@chu-nice.fr (A.I.)

**Keywords:** body mass index, job-related stress, night shift work, obesity, occupational health, sedentary lifestyle, socio-professional disparities, tertiary sector, weight gain, workplace health interventions

## Abstract

**Background/Objectives:** Work environments play a crucial role in shaping lifestyle behaviors that influence body weight, yet the relationship between occupational factors and obesity remains underexplored. This study assessed the impact of work-related conditions on body mass index (BMI) trends in a large cohort of tertiary sector employees in France. **Methods**: A retrospective observational analysis was conducted using occupational health data from 23,853 employees in Paris. BMI changes were assessed through linear regression models, and associations between occupational exposures (e.g., night work, sedentary roles) and BMI variation were examined. **Results**: A total of 23,853 employees were analyzed. The mean age at first visit was 45.4 years (range 16–82), and 59% were women. Employees belonged to various socio-professional categories, with more than half in executive or intermediate positions. At baseline, 24% were overweight and 8.5% obese. Mean BMI was 23.5 kg/m^2^ in women and 24.7 kg/m^2^ in men, with average annual increases of 0.15 and 0.12 kg/m^2^, respectively (*p* < 0.05). **Conclusions**: Work-related factors, particularly night shifts, sedentary roles, and lower occupational status, contribute to BMI increases among tertiary sector employees.

## 1. Introduction

Over the past two decades, the prevalence of obesity has risen sharply in France, affecting 17% of adults in 2020 compared to just 6.1% in 1980 [1,2]. This multifactorial condition results from a complex interplay of genetic and environmental factors. Dietary changes and reduced physical activity are well-established contributors, driven by larger portion sizes, higher food energy density, increased availability, and price fluctuations [3,4,5,6]. Additionally, modern sedentary lifestyles, marked by prolonged screen time, video games, and reliance on cars and public transport, have significantly reduced energy expenditure. Other environmental and behavioral factors, such as chronic stress, sleep disturbances, certain medications, gut microbiota composition, and exposure to pollutants, further exacerbate obesity risk [6].

Body mass index (BMI), defined as weight in kilograms divided by the square of height in meters (kg/m^2^), is the most widely used indicator for classifying underweight, overweight, and obesity [1]. Beyond being a simple anthropometric index, BMI correlates with adiposity and with the risk of obesity-related complications such as cardiovascular disease, type 2 diabetes, and premature mortality [3,4,5]. For this reason, BMI is central to epidemiological studies and to occupational health surveillance, where it provides a practical measure to monitor weight trajectories across large populations.

The relationship between obesity and work is complex and bidirectional. On the one hand, obesity can be a professional disadvantage, limiting employment opportunities and affecting productivity. On the other, specific work conditions, such as prolonged sedentary behavior, chronic stress, and shift work, can promote weight gain and increase the risk of obesity [7]. This phenomenon, characteristic of modern societies, raises concerns about evolving work environments that are increasingly sedentary and stressful. Moreover, the growing prevalence of obesity in the workplace generates substantial economic costs due to increased absenteeism and healthcare expenditure [8,9].

Workplace exposures such as sedentary roles, irregular or night shifts, psychological stress, and low job control have all been associated with weight gain [7,10,11,12]. In addition, work schedules interfering with sleep and circadian rhythms may alter hormonal regulation of appetite and energy balance, thereby increasing the risk of obesity [13,14,15,16,17,18]. These occupational factors can thus act synergistically with lifestyle determinants, amplifying weight gain among vulnerable workers.

As in the general population, the prevalence of obesity continues to rise among workers. However, this increase is more pronounced among manual laborers and agricultural workers compared to executives and professionals, widening the gap between socioeconomic groups [10]. In France, this gradient reflects not only differences in income and education but also the unequal distribution of occupational risks such as shift work and job strain [2,19]. These disparities highlight the need for workplace-level interventions and for preventive strategies that specifically address high-risk employee groups.

Given this alarming trend, preventing obesity and identifying at-risk individuals have become critical public health priorities [9]. In this context, analyzing BMI trends in relation to occupational exposures is essential for designing effective workplace health policies. The present study investigated a large cohort of tertiary-sector employees in France, with the objective of identifying work-related risk factors influencing BMI. By quantifying the association between professional conditions, socio-professional categories, and BMI progression, our work aims to provide evidence to guide targeted prevention and health promotion strategies in the occupational setting.

## 2. Materials and Methods

### 2.1. Study Design

This retrospective, observational study was based on data from employees in the tertiary sector in France, collected between 1 January 2005 and 31 December 2015, by physicians at the Centre Médical Bourse (CMB). The latter is an inter-company occupational health service governed by the French Labor Code, located in Paris (France). The CMB’s multidisciplinary team advises and supports employers in the tertiary sector on the implementation of occupational risk prevention and monitors the occupational health of their employees. The CMB also aims to develop prevention actions in the workplace and information about occupational risks to raise the awareness of employers and employees.

The CMB uses specially designed software to collect and record employee data at each doctor visit as part of routine occupational health monitoring. The encrypted data, including age, weight, BMI, waist circumference, and seniority, must be entered manually, as well as certain non-encrypted data, such as sex, contract, and type of job. To ensure privacy, each employee was identified by an alphanumeric code. The data extracted from the software were divided into several sections: the number of visits by each employee; the average weight and average BMI recorded during the different visits; the BMI coefficient (kg/m^2^/year), that is, the linear regression of BMI according to the examination dates of the different visits; the socio-professional category (SPC), and the working characteristics. With regard to the SPC section, the classification was the following: senior manager (A), executive (B), intermediate (C), and master/supervisor (D). The working characteristics were categorized according to the following: day/night work; function with strong human responsibility; functional organization of the activity; physically heavy work; work requiring road travel; thermal environment work with the risk of physical or verbal aggression; mental load; work requiring a long business trip; repetitive movements of the upper limbs; prolonged sitting posture; display screen; phone call; standing posture; emotionally charged work; remote working; displacement disrupting chronobiology (time difference); work under time pressure; handling; and regular contact with the public.

### 2.2. Study Objectives

The primary objective was to study the change in BMI between the medical consultations each employee had at the CMB during the period considered. Secondary objectives were to investigate the relationship between working conditions and BMI, the association between socio-professional categories and BMI, and identified employee profiles at risk of obesity.

**Figure 1:**
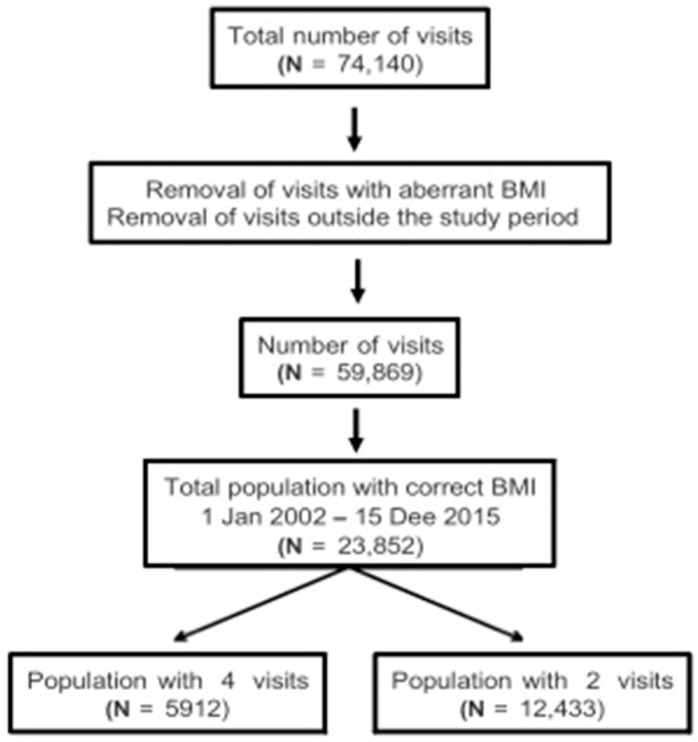
Flow chart of the study population.

In order to show changes in BMI, the trend or linear regression between at least two BMIs was calculated; hence, from at least two medical consultations. Two subgroups of employees were created based on their number of visits: group 1 included employees who had had at least two visits, and group 2 included those who had had at least four visits. Employees with fewer than 2 visits were excluded from the study. A flow chart of the study population is shown in Figure 1.

Moreover, in order to assess the knowledge and degree of motivation for the management of obesity by CMB doctors, a 10-question questionnaire was created on the SurveyMonkey site (Supplementary Scheme S1). The questionnaire was sent to the 19 CMB doctors and 100% of the doctors replied.

### 2.3. Statistical Analysis

Employees were eligible if they had at least two recorded occupational health visits between 2005 and 2015 with complete anthropometric data (age, sex, height, weight). Those with only one visit or with missing essential data were excluded. To ensure robust estimation of BMI change over time, subgroup analyses were conducted among employees with ≥4 visits. No formal a priori sample size calculation was performed; instead, all eligible employees from the occupational health database during the study period (*n* = 23,853) were included, thus maximizing statistical power and external validity.

All data were manually entered by occupational physicians into the DYNAMIT^®^ (2004 version) software and subsequently verified and cleaned before analysis. Implausible BMI values (<13 kg/m^2^ or >60 kg/m^2^) and the corresponding visits were excluded prior to computation of individual BMI slopes. Extreme BMI slope values (e.g., >20 or <−15 kg/m^2^/year) occasionally appeared when large BMI differences occurred between a small number of visits. These cases represented less than 0.5% of all records and were retained because their inclusion had a negligible impact on overall means and significance levels. No additional sensitivity analyses could be performed, as the anonymized dataset is no longer accessible.

To calculate the rate of change of BMI/year (BMI coefficient) of a given employee, linear regression was applied to the data corresponding to all the visits of the employee. This linear regression aimed to evaluate the BMI change according to the visit dates, making it possible to find the trend in BMI evolution in kg/m^2^/year for employees with two or more visits. Employees with only one visit had zero linear regression. Being a linear regression using the least squares method with a single explanatory variable, the result is a straight line (*y* = *mx* + *b*) where the parameters of the affine function are given by the following formulae:

$$m=\frac{\sum_{i=1}^{n}\left(X_{i}-\bar{X})(Y_{i}-\bar{Y}\right)}{\sum_{i=1}^{n}(X_{i}-\bar{X}{)}^{2}}\;\text{and}\;b=\bar{Y}-m\bar{X}$$

(*m*: rate of change in BMI/year; *i*: one visit; *n*: number of visits; *X*_*i*_: date of visit expressed in years; *Y*_*i*_: BMI of the employee during the visit; $\bar{X}$: average of visits; $\bar{Y}$: mean BMI).

Continuous demographic variables are expressed as mean ± standard deviation (SD) and range (min–max). Categorical variables are reported as number and percentage. Continuous outcome variables are generally reported as mean ± SD, and range. The Student’s *t*-test was used to measure the difference between two mean rates of change in BMI/year with different variances. To identify the different factors implicated in the definition of the BMI coefficient, the data were analyzed from different angles: men vs. women; across categories; day work vs. night work; and across BMI groups (underweight, normal, overweight, obese). The following were also considered: men/women and categories; men/women and day/night work; categories and day/night work; men/women, categories, and day/night work. Sectors of activity and exhibitions were studied separately. A one-way ANOVA for independent measures was used to compare the different socio-economic categories. Post hoc pairwise comparisons were performed using Tukey’s HSD test following significant one-way ANOVA results. Night work was treated as a fixed categorical variable, defined according to the job title or exposure descriptors recorded in the DYNAMIT^®^ software at each medical visit. The database did not provide longitudinal tracking of transitions between day and night schedules, and potential changes in work patterns over time could not be analyzed. Results are presented as mean ± SD and exact *p*-values, reflecting the descriptive purpose of the analysis. A *p* value < 0.05 was considered statistically significant.

## 3. Results

### 3.1. Study Population

The total number of employees analyzed during the study period was 23,853. Group 1 included 12,433 employees who had had at least two visits and group 2 included 5912 employees who had had at least four visits. The majority of the 12,433 employees identified with two or more visits were women (59%). The main characteristics studied in this patient population are summarized in Table 1.

In group 1 of employees, the mean BMI was 23.5 kg/m^2^ (range: 15.1–53.7) for women and 24.7 kg/m^2^ (range: 15.2–55.7) for men, with a mean rate of change in BMI of 0.15 kg/m^2^/year for women and 0.12 kg/m^2^/year for men (*p* = 0.017). For employees who had four or more visits (*n* = 5912), the mean age was 48.4 years and the mean number of visits was 5.6, with a mean time of 1.6 years between two visits. The mean BMI in women was 23.6 kg/m^2^ vs. 24.8 kg/m^2^ in men (*p* = 0.016). The mean rate of change in BMI in women was significantly higher than that in men (0.14 kg/m^2^/year vs. 0.11 kg/m^2^/year; *p* = 0.021). With a mean of 5.6 consultations per employee at the CMB, the mean rate of change in BMI/year was 0.13 (Table 1).

### 3.2. Socio-Professional Categories, Sectors of Activity, and BMI

In both groups of employees, more than half of the population studied belonged to the manager or intermediate categories. The rate of increase in BMI was inversely proportional to the socio-professional category, and this was independent of sex, even if it seemed more marked in women (Table 2). Notably, subjects in Category A were significantly older (*p* = 0.020) and exhibited both a lower baseline BMI (*p* = 0.012) and a slower rate of BMI increase than those in Category D (*p* = 0.011).

Concerning the different types of occupational exposure, night work and call center duty significantly increased the rate of change in BMI compared to other employees (0.18 vs. 0.13 kg/m^2^/year *p* = 0.013, and 0.25 vs. 0.13 kg/m^2^/year; *p* = 0.011). Seventy percent of people who worked in the call center were women, with a significantly higher rate of increase in BMI compared to men (0.3 kg/m^2^/year vs. 0.12 kg/m^2^/year; *p* < 0.05). Meanwhile, protective factors were jobs needing professional travel, occupations with imposed time limits, functions with high human responsibility, and jobs with the need to carry heavy loads (Table 3).

### 3.3. Night Work and BMI

Of the total female employee sample with two or more visits (*n* = 7370), only 1.3% were working at night (*n* = 96). In this subgroup, the mean age was 41 years, while it was 45 years among female day workers (*p* = 0.033). The mean rate of change in BMI/year was 0.14 among female day workers (*n* = 7274) vs. 0.29 among night workers (*n* = 96) (*p* = 0.078). Among men (*n* = 5063), the proportion of employees working at night was higher (*n* = 419, 8.2%) with a lesser a priori impact on BMI (0.16 vs. 0.12 kg/m^2^/year for day workers, *p* = 0.098).

**Figure 2:**
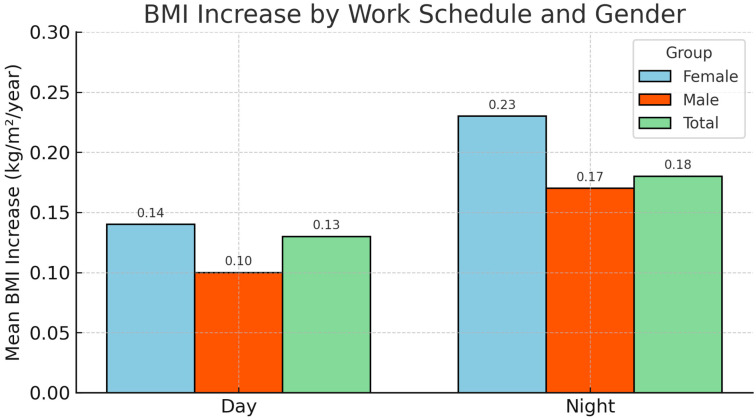
Mean increase in BMI (kg/m^2^/year) in day workers vs. night workers having more than four visits to the CMB.

Among the employees who had four or more visits to the CMB, 5647 employees worked during the day (96%) and 265 employees worked at night (4.5%). On average, day workers had fewer visits than night workers (5.54 vs. 6.13, respectively; *p* = 0.034). Day workers had a significantly lower weight (68.6 kg), a lower average BMI (24.1 kg/m^2^), and a lower mean rate of change in BMI/year (0.13 kg/m^2^/year) than night workers (75.2 kg, 25 kg/m^2^, and 0.18 kg/m^2^/year; *p* = 0.011; *p* = 0.012; *p* = 0.016). Among women, there were 3579 working day employees and 48 working nights (1.3%). The mean BMI/year change rates were higher among night workers than day workers, but the difference was not significant (0.23 vs. 0.14 kg/m^2^/year; *p* = 0.61). Among men, there were 2068 employees working during the day and 217 working at night (9.5%). The mean rate of change in BMI/year was higher among night workers than day workers (0.17 vs. 0.10 kg/m^2^/year; *p* = 0.010) (Figure 2).

### 3.4. Overweight and Obesity Among Employees

**Figure 3:**
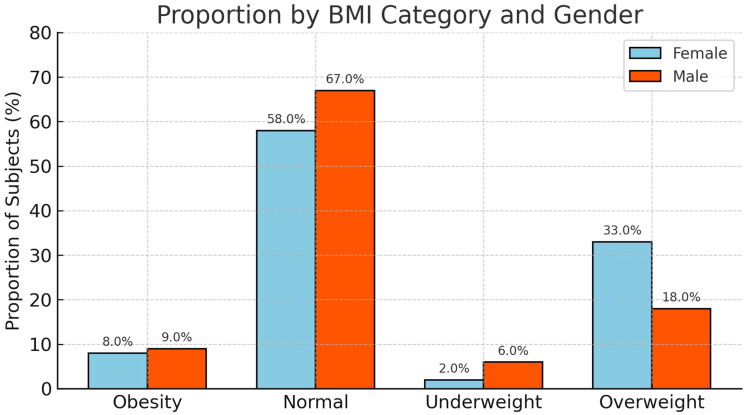
Proportion of subjects with more than two visits to the CMB in each BMI category.

In the population of employees with two or more visits, 24% (*n* = 2982) were overweight and 8.5% (*n* = 1052) obese, and 24% (*n* = 252) of these had a BMI ≥ 35 (severe or morbid obesity) (Figure 3). The BMI coefficient was significantly higher for patients who were overweight or obese (0.19 kg/m^2^/year vs. 0.11 kg/m^2^/year for BMI < 25; *p* < 0.001). In the subgroup of employees working at night, the rate of increase in BMI was greater than that of day workers, and this difference was accentuated for groups with a BMI > 25 kg/m^2^ (0.22 kg/m^2^/year vs. 0.16 kg/m^2^/year for BMI > 25; *p* < 0.001). Considering the results by sex, among overweight woman (BMI between 25 and 30 Kg/m^2^) working at night, the rate of increase in BMI was more than double compared to overweight men working at night (0.45 kg/m^2^/year vs. 0.19 kg/m^2^/year).

In the population of employees with four or more visits, 25% of patients had a BMI > 25 kg/m^2^ (*n* = 1483), and 8.7% (*n* = 517) had a BMI > 30 kg/m^2^. In this group of employees, the mean rate of increase in BMI was higher among night workers who were overweight (n. 95) and obese (n. 25) compared to day workers (n. 1388, and n. 492) with the same BMI (0.24 kg/m^2^/year and 0.48 kg/m^2^/year vs. 0.17 kg/m^2^/year and 0.22 kg/m^2^/year, respectively; *p* = 0.011 and *p* = 0.031). Moreover, female employees (n. 681) who were overweight showed a significantly higher increase in BMI coefficient than male employees (n. 787) with the same BMI (0.24 vs. 0.11 kg/m^2^/year; *p* < 0.001).

### 3.5. Online Questionnaire

Concerning the online questionnaire answers, the results showed how the participating occupational medicine doctors were globally aware of the obesity problem. In particular, they almost always calculated the patient’s BMI and discussed with the patient the possible solutions to be undertaken to treat the disease (Supplementary Scheme S1).

## 4. Discussion

Multiple studies have already established a correlation between work activity and obesity. In particular, factors such as professional position, occupational stress, night work, and sedentary work can lead to changes in eating behavior, a disorganization of eating patterns, a marked reduction in physical activity, and an alteration of the duration and quality of sleep, which all contribute to weight gain [10,11,12].

The present study focused on the possible correlations between night work and BMI increase. According to many studies, the temporal disorganization induced by the succession of shifts at alternating times has important consequences for the health of the worker, including obesity [13,14]. Alteration of the physiological sleep cycle seems to be one of the possible explanations. In particular, this alteration leads to so-called sleep debt, since daytime sleep following a night shift is on average shorter and of worse quality. In the present cohort of employees, objective data concerning the repercussions of shift work and night work on BMI and the relationship between BMI and the different socio-professional categories were studied. The initial sample of 12,433 employees, with a mean number of occupational health visits of less than four, did not allow us to highlight a significant difference in the rate of BMI variation over time as a correlation of the employees’ time constraints and professional categories. A subgroup of these employees who had at least four visits to occupational medicine was also studied. Since the mean time between two visits was 1.6 years, it was possible to outline more precisely the BMI change patterns and determine the factors underlying these changes. It emerged that night work and call center or switchboard activity (for example, in hotels or hospitals) were associated with a statistically significant rate of increase in BMI.

These results are consistent with other studies regarding the correlation between night work and obesity. Marquezea et al. recorded a BMI coefficient of 0.24 kg/m^2^/year for night shift nurses, although total daily sleep duration was greater than for day work staff [15]. Rohmer et al. also found a direct correlation between night work and weight gain, even though the motor activity of night work nurses who were obese was higher than those who worked during the day [16]. According to these studies, the weight gain linked to night work would not appear to be linked solely to the amount of sleep or to the physical activity performed; rather, it could depend on much more complex hormonal mechanisms regulated by the circadian rhythm. Concerning this factor, several studies suggest a correlation between the desynchronization of circadian rhythm and alterations in metabolic regulatory network, in particular, lipid homeostatic genes, which could lead to an increase in BMI [17]. Moreover, Scheer et al. discovered in 2009 that a change in the circadian rhythm leads to a decrease in leptin secretion (a hormone involved in the feeling of satiety), and the importance of the decrease was directly linked to the desynchronization duration. This hormonal imbalance, in reducing satiety, could lead to a change in eating behavior for night workers, increasing their tendency to snack [18].

According to the French Agency for Food, Environmental and Occupational Health Safety (ANSES) report, the population involved in night work, whether usual or occasional, has almost doubled in 20 years. In 2012, it represented 15.4% of employees (i.e., 3.5 million people) and this continues to increase [19]. Given the close link between night work and health, night workers are currently subject to enhanced medical monitoring (4.3 visits on average vs. 3.8 for day workers) in the CMB. Our results seem to support this attitude. By stratifying day and night workers according to their BMI, the study demonstrated a significant increase in the BMI coefficient of overweight night workers and especially in obese workers (0.24 kg/m^2^/year vs. 0.48 kg/m^2^/year among day workers). It can be deduced that in employees who are already overweight or obese, a desynchronization of their circadian rhythm would increase the rate of weight gain. Our findings may suggest the need for targeted workplace prevention programs for individuals with higher BMI working night shifts, particularly in the tertiary sector.

Furthermore, in this population sample of tertiary sector workers in the Paris region, despite the existence of various biases (lifestyle, social status, ethnic origin, age, smoking or alcohol consumption, family history of obesity, diet, and stress level), the results of this study are consistent with the French National Institute of Statistics and Economic Studies (INSEE) report in 2007 [20], which highlighted that the gap between the different social categories with regard to obesity had increased. In the present study, a significantly lower coefficient of increase in BMI was observed in upper-management employees than in supervisors (0.09 vs. 0.15 kg/m^2^/year). Education level and salary level therefore seem to have a direct effect on BMI.

In the general population, the prevalence of obesity is higher in women (17.6%) than in men (16.1%). It can be doubled or even tripled in underprivileged areas, particularly among women [1,2]. These results can be partly explained by the different eating behavior of women, less physical activity, successive pregnancies, and/or a higher proportion of women in precarious situations or with sedentary jobs. This phenomenon can be illustrated by considering the employees in the current study declaring on-call duty: 70% of them were women, and the rate of increase in their BMI was significantly higher than that of men (0.12 kg/m^2^/year vs. 0.3 kg/m^2^/year). In this study, about 9% of the population studied were obese (BMI ≥ 30 kg/m^2^), and the majority were women, unlike the category of overweight employees (24%), where the majority were men. Female employees who were overweight had a significantly higher increase in their BMI coefficient than men (0.24 kg/m^2^/year vs. 0.11 kg/m^2^/year). This difference was not recorded for the other BMI categories. For this reason, a therapeutic education program for women at risk of progressing from overweight to obesity would be more than justified, as proposed by Sanguignol et al., to motivate long-term behavior change [21].

This study has several limitations. First, its retrospective design and reliance on routinely collected occupational health data may have introduced selection and information bias. Second, several important covariates are absent from the occupational health database. Information on leisure-time physical activity, dietary habits, smoking status, alcohol consumption, and use of weight-modifying medications (e.g., antidepressants, corticosteroids) was not available. Similarly, major medical interventions that could affect body weight (such as bariatric surgery or long-term hospitalization) could not be identified from the data and therefore were not excluded from the analyses. Furthermore, family history of obesity and detailed socioeconomic indicators (such as income or educational attainment beyond socio-professional category) were lacking. Third, while work schedules (day vs. night) were recorded, the database did not allow us to reliably capture job changes or transitions to and from night work over time. These missing data may have introduced residual confounding and limit the ability to fully disentangle occupational from lifestyle determinants of BMI trajectories. Forth, although several between-group differences reached statistical significance, their absolute magnitude was small and should be interpreted as of limited clinical relevance. The analyses were exploratory and designed to identify associations rather than infer causality. Comparisons by sex, socio-professional category, and work schedule were pre-specified in the study design. Because of the descriptive and hypothesis-generating nature of the study, no formal correction for multiple testing was applied, and this limitation should be taken into account when interpreting the results. Finally, the study population was limited to tertiary-sector employees in the Paris region, which may reduce generalizability. Future prospective studies should systematically collect such variables to provide a more comprehensive understanding of the relationship between work, lifestyle, and obesity risk. Moreover, our two-step approach—computing individual BMI slopes and comparing group means—facilitates interpretation but may be statistically less efficient than mixed-effects models. Because the anonymized data extracted from the occupational health software are no longer accessible, additional modeling could not be performed. Future work using reconstituted or prospective datasets should implement mixed-effects analyses to refine these results.

This study is based on data collected by occupational physicians who have to evaluate different aspects of employee health. For this reason, a lack of knowledge of the obesity problem and its impact on the quality of work would have reduced the reliability of the data, constituting an important bias in the study. According to the results of the online questionnaire sent to the CMB doctors, it appears that they are generally aware of the problem of obesity. The majority (73.6%) expressed a desire to take care of the affected employees by setting up a specialized care network. In the event of morbid obesity (2% of the initial sample of this study), the doctor should be able to offer the employee multidisciplinary care that could ultimately lead to bariatric surgery. On the other hands, the online questionnaire sent to the occupational physicians was created by the study team and pilot-tested internally by several physicians to ensure clarity and feasibility. However, it was not externally validated against standardized instruments, which constitutes a limitation. Future studies should use validated tools to assess physicians’ knowledge and practices regarding obesity management.

## 5. Conclusions

Obesity is influenced by multiple factors, including work-related conditions. This study shows that BMI increases similarly in men and women, but at a faster rate in lower socio-professional categories. Night work and call center duty significantly contribute to weight gain, particularly in employees already overweight or obese. These high-risk individuals should receive targeted support, including psychological, nutritional, and physical coaching. Occupational health programs should systematically track BMI to better identify at-risk employees and justify closer monitoring of night workers.

## Figures and Tables

**Table 1:** Characteristics of the study population with ≥2 and ≥4 visits to the CMB.

Variable	≥2 Visits–Females (*n* = 7370)	≥2 Visits–Males (*n* = 5063)	≥4 Visits–Females (*n* = 3627)	≥4 Visits–Males (*n* = 2285)
Sex (%)	59.3	40.7	61.3	38.7
Number of visits to the CMB	3.9 ± 1.9 (2–19)	3.8 ± 2.0 (2–17)	5.6 ± 1.5 (4–19)	5.6 ± 1.6 (4–17)
Age at first visit (years)	45.5 ± 10.1 (16–79)	45.2 ± 10.3 (17–82)	48.2 ± 9.0 (26–79)	48.6 ± 9.1 (18–76)
BMI (kg/m^2^)	23.5 ± 4.5 (15.1–53.7)	24.7 ± 3.6 (15.2–55.7)	23.6 ± 4.4 (15.6–49.7)	24.8 ± 3.6 (15.2–55.7)
Rate of BMI increase (kg/m^2^/year)	0.15 ± 0.8 (–15.73 to 22.16)	0.12 ± 0.5 (–5.11 to 5.51)	0.14 ± 0.4 (–3.59 to 3.03)	0.11 ± 0.3 (–3.12 to 1.72)

All values are expressed as mean ± standard deviation (SD), with range in parentheses. *p*-value for females vs. males:–Rate of BMI increase: *p* = 0.017 (≥2 visits), *p* = 0.021 (≥4 visits).

**Table 2:** Weight gain according to socio-professional category (≥4 visits to the CMB).

Variable	Category A (Senior Executive)	Category B (Executive)	Category C (Intermediate)	Category D (Low Qualification)
Category (%)/N	8.5/1056	39.5/4919	34.2/4258	17.7/2200
Number of visits	5.3 ± 1.5 (4–13)	5.4 ± 1.4 (4–12)	5.6 ± 1.5 (4–17)	5.9 ± 1.8 (4–19)
Age at first visit (years)	52.6 ± 7.8 (29–71)	47.3 ± 8.9 (26–76)	47.9 ± 8.9 (27–78)	49.8 ± 9.3 (18–79)
BMI (kg/m^2^)	24.3 ± 3.8 (17.2–39.7)	23.7 ± 4.0 (15.6–49.7)	23.9 ± 4.3 (15.8–48.6)	25.0 ± 4.3 (15.2–55.7)
Rate of BMI increase (kg/m^2^/year)	0.09 ± 0.33 (–1.81 to 3.03)	0.12 ± 0.33 (–3.59 to 2.18)	0.13 ± 0.38 (–31.2 to 2.98) *	0.15 ± 0.37 (–31.5 to 2.26) *

All values are expressed as mean ± standard deviation (SD), with range in parentheses. *p*-values from ANOVA for independent measures: Number of visits: *p* = 0.042. Age at first visit: *p* = 0.031. BMI: *p* = 0.032. Rate of BMI increase: *p* = 0.022. * Due to data access limitations, median or truncated values could not be calculated. Extreme values (>20 or <−15 kg/m^2^/year) represented <0.5% of records (Category C: n.16, 0.37%; Cat D n.10, 0.45%).

**Table 3:** List of occupations significantly associated with weight gain or protective occupations.

Occupation	Mean Rate of BMI Increase (kg/m^2^/year)	Rest of Population (kg/m^2^/year)	*p*-Value
Night work (*n* = 265)	0.18	0.13	**0.013**
Telephone-based work (*n* = 90) **	0.25	0.13	**0.011**
Professional travel (*n* = 825)	0.10	0.13	**0.034**
Work with time constraints (*n* = 48) **	0.08	0.13	**0.022**
Carrying heavy loads (*n* = 26) **	−0.04	0.13	**0.011**
Jobs with human responsibility (*n* = 16) **	0.06	0.13	**0.022**

*p*-values in bold indicate statistically significant risk factors for weight gain. ** these subgroup findings are exploratory due to the small number of subjects in each group.

## Data Availability

The raw individual-level data are no longer accessible due to CNIL regulations and the expiration of authorization. However, aggregated summary data and a dictionary of variables used in the analysis can be shared with authorized researchers upon formal request, pending approval by the CNIL and relevant ethical committees.

## Supplementary Materials

The following supporting information can be downloaded at https://www.mdpi.com/article/10.3390/jcm14207399/s1, Scheme S1: Questionnaire sent to the BMC practitioners and response results.
