# Supplementary material for: Work-Related Factors and Their Influence on Body Mass Index: A Retrospective Cohort Study in the French Tertiary Sector
Source: J Clin Med. 2025 Oct 20;14(20):7399. doi: 10.3390/jcm14207399 (PMC12565228; doi:10.3390/jcm14207399)
Supplement: Supplementary file 1 [file jcm-14-07399-s001.zip › jcm-3894780-supplementary.pdf]

Supplementary Scheme 1. Questionnaire sent to the BMC practitioners

Question

1. **How long have you worked as a physician in occupational health?**
   - < 1 year (R: 5.26% N=1)
   - 1–2 years. (R: 5.26% N=1)
   - 2–5 years. (R: 5.26% N=1)
   - 5–10 years. (R: 10.53% N=2)
   - 10–20 years. (R: 26.32% N=5)
   - >20 years. (R: 47.37% N=9)
2. **According to you, what is the prevalence of obesity in France?**
   - Approximately 5%. (R: 15,.79% N=3)
   - Approximately 15%. (R: 63.16% N=12)
   - Approximately 25%. (R: 21.05% N=4)
3. **According to the HAS, above what BMI does global mortality increase (except for elderly subjects)?**
   - BMI = 28 kg/m$^{2.}$ (R: 63.16% N=12)
   - BMI = 38 kg/m$^{2.}$ (R: 36.84% N=è7)
   - BMI = 48 kg/m$^{2.}$ (R: 0% N=0)
4. **How often do you measure BMI in the workers that you see in consultation?**
   - Never (R: 5.26% N=1)
   - 20% of consultations (R: 5.26% N=1)
   - 20–40% of consultations. (R: 0% N=0)
   - 40–60% of consultations (R: 5.26% N=1)
   - 60–80% of consultations (R: 5.26% N=1)
   - 80–100% of consultations (R: 78.95% N=15)
5. **From what BMI do you consider a subject to be obese?**
   - BMI ≥25 kg/m^2 (R: 5.26% N=1)
   - BMI ≥30 kg/m^2 (R: 89.47% N=17)
   - BMI ≥40 kg/m^2 (R: 5.26% N=1)
6. **From what BMI do you consider obesity to be 'massive' or 'morbid'?**
   - BMI ≥35 kg/m^2 (R: 36.84% N=7)
   - BMI ≥40 kg/m^2 (R: 57.89% N=11)
   - BMI ≥45 kg/m^2 (R: 5.26% N=1)

7. **In most cases, what is the problem/subject linked to obesity that you deal with most often in your obese workers (multiple choice)?**
   - ☐ Somatic complications linked to obesity (diabetes, cardiovascular, cutaneous, respiratory, cancers, etc.) (R: 78.95% N=15)
   - ☐ Problems of integration in professional life (R: 15.79% N=3)
   - ☐ Aesthetic problems (R: 10.53% N=2)
   - ☐ Psychological problems (R: 42.11% N=8)
   - ☐ Possible solutions to tackle obesity (R: 94.74% N=18)
   - ☐ Approaches that the worker has already put in place to manage their obesity (R: 94.74% N=18)
   - ☐ None. (R: 0% N=0)
8. **If you have access to a network for the management of obesity by bariatric surgery (including nutritional advice, pre- and post-operative psychological follow-up), would you recommend bariatric surgery to workers with a BMI $\geq$40 kg/m^2?**
   - ☐ Yes (R: 68.42% N=13)
   - ☐ No (R: 31.58% N=6)
9. **Do you know of one or more networks for the management of obesity and have you already used them?**
   - ☐ Yes, and I have already used one of these networks. (R: 31.58% N=6)
   - ☐ Yes, but I have never used one of these networks. (R: 21.05% N=4)
   - ☐ No (R: 47.37% N=9)

10. **Would you be in favour of the introduction of a 'structure for dietetic follow-up and work' within the Centre Médical Bourse?**
   - ☐ Yes (R: 73.68% N=14)
   - ☐ No. (R: 26.32% N=5)
